# Chemical Properties of Peanut Oil from *Arachis hypogaea* L. ‘Tainan 14’ and Its Oxidized Volatile Formation

**DOI:** 10.3390/molecules27206811

**Published:** 2022-10-11

**Authors:** Kai-Min Yang, Ming-Ching Cheng, Zih-Sian Ye, Lee-Ping Chu, Hsin-Chun Chen

**Affiliations:** 1 Department of Food Science, National Quemoy University, Kinmen 892, Taiwan; 2 Department of Health Food, Chung Chou University of Science and Technology, Changhua 510, Taiwan; 3 Department of Cosmeceutics, China Medical University, Taichung 406, Taiwan; 4 Department of Orthopedics, China Medical University Hospital, Taichung 404, Taiwan

**Keywords:** peanut oil, oxidative stability, volatile

## Abstract

*Arachis hypogaea* L. ‘Tainan 14’ has purple skin characteristics. This study investigated the effects of different materials (shelled or unshelled peanuts) and temperatures (120 or 140 °C) on the properties of extracted peanut oil. The results show that its antioxidant components (total flavonoid, α–tocopherol, and γ-tocopherol) and oxidative stability were mainly affected by the roasting temperature (*p* < 0.05). Fifty-eight volatile compounds were identified by peanut oil oxidation and divided into three main groups during the roasting process using principal component analysis. The volatile formation changes of different materials and temperatures were assessed by agglomerative hierarchical clustering analysis. These results provide useful reference information for peanut oil applications in the food industry.

## 1. Introduction

The global production and consumption of major edible oils are increasing annually. People’s eating habits have changed from being rich in plant-derived oil to being rich in animal-derived oil [1,2,3]. Palm, soybean, and rapeseed oil remain the three primary edible oils globally [2]. Currently, the global production of edible vegetable oils is 200 million tons, projected to increase by over 40 million tons by 2025 [3]. Edible oils are a vital part of the human diet, contributing to food’s increased palatability and satiated feelings [4,5]. In addition, they provide essential fatty acids and energy, serve as the carrier for fat-soluble nutrients, and are a precursor for hormone and prostaglandin synthesis [6,7]. Edible oils play a major role in various cooking techniques, from sautéing and frying to roasting and baking. Not all edible oils are heat stable enough or intended for use in cooking, the key issue being their smoke point. The smoke point refers to when the oil stops shining and begins smoking [8,9]. Cooking food in oil past its smoke point creates a burned, charred taste that is unpleasant. It also negates all the oil’s healthy nutrients and phytochemicals, which are destroyed when it becomes too hot [8].

Relevant research on Chinese daily edible oil intake (34 g) found it to be mostly higher than the recommended amount (30 g) [10]. However, several chronic or non-infectious diseases are caused by the high consumption of edible oils. Edible vegetable oils are obtained by two methods: cold-pressed only and solvent extraction with preliminary treatment. Cold-pressed oils are rich in antioxidants and bioactive substances; they benefit nutrition and health [11,12,13]. In addition, the extraction of cold-pressed oils does not involve chemical contact and is considered as a safer product [13]. Most studies have evaluated the nutritional value of edible oils by fatty acids and unsaponifiables. From nutritional and health-promoting perspectives, cold-pressed oil is rich in antioxidants and bioactive substances such as carotenoids, vitamin A, vitamin E, phytosterols, and polyphenols [11,12,13,14]. Unsaponifiables have health-promoting effects and improve oil stability including sesame in sesame oil, polyphenols in olive oil, kaempferol in camellia oil, and γ-oryzanol in rice bran oil [15,16,17,18]. The peanut (*Arachis hypogaea*) is a plant of the Fabaceae family (legumes) and is an excellent source of oil (50%), protein (approximately 25% of energy), and dietary fiber (9%) [19]. Peanuts are the most important oil-bearing industrial crop, with worldwide production reaching 43.98 million tons [20]. Oil and food production are the two main uses of peanuts. Cold-pressed peanut oil is the traditional bulk edible oil in China and India, which is suitable for deep frying foods due to its high smoke point and low production of *trans* fatty acids [19,20]. Peanut oil has a unique flavor, which is very popular with numerous consumers. The roasting technique can significantly affect the volatile components, changing the flavor of plant oils from a somewhat grassy, mild, sweet, and desirable aroma to an irritating, scorched smell [21,22,23].

Peanut composition differs greatly due to the improved crop variety [19]. In this study, Tainan 14 peanuts were selected due to their unique characteristics including purple skin, which has a higher polyphenol content than normal brown peanuts. The peanut oil production process is simple, but it varies by region. For example, the raw material can be shelled or unhulled peanuts, impacting the processing simplicity, hygiene, sensory, and storage stability. Furthermore, roasting is a critical process. Therefore, this study on peanuts oil focused on different raw materials (shelled or unshelled) and temperatures (120 or 140 °C) to better understand the chemical properties related to the oil’s quality. In particular, we (1) compared the characteristics of peanut oil with the values established by Codex Alimentarius; (2) evaluated its antioxidant properties and oxidative stabilities to provide insight into the functional oil; and (3) evaluated the peanut oils by the Rancimat test and observed changes in their volatile composition.

## 2. Results

### 2.1. Chemical Property Changes

Producing vegetable oils by mechanical pressing is inefficient but represents a green technology [11]. The results show that the P12, P14, HP12, and HP14 yields were 32.55%, 33.61%, 29.78%, and 30.13%, respectively (Table 1). Other studies have shown that high roasting temperature promotes the browning reaction. However, this study shows that the oil’s appearance did not change significantly (browning index, BI: 25.67–26.63) by roasting at 120 or 140 °C (Table 1).

The degree of oxidation was influenced by roasting temperature based on the oxidation rate. Lipid oxidation requires more than one assay to monitor the reaction since a single assay cannot measure the whole process, nor can it be equally useful at all oxidation stages [24,25,26]. Peroxide value (PV), acid value (AV), thiobarbituric acid-reactive substances, and conjugated dienes and trienes are standard AOCS-approved methods. Peanut oils produced with different materials and roasting temperatures had AVs of 0.98–1.65 mg potassium hydroxide/g, PVs of 5.61–6.88 mEq oxygen/kg, and (p)-anisidine value (p-AV) of 6.15–12.63 mEq/kg. These results were confirmed by the Codex Alimentarius standard. Previous studies have shown that PV and p-AV can be used to evaluate the primary and secondary oxidation products, respectively. The degree of oxidation can be determined by converting PV and p-AV to a total oxidation value (TOTOX), which has industry-approved limits for fish oil (26) and edible oil (30) according to the standard [25]. The results showed that the TOTOX values for P12, P14, HP12, and HP14 were 17.35, 24.86, 20.63, and 24.42, respectively.

We identified eight fatty acids in peanut oil (Table 1) that were, in decreasing concentration: linoleic acid, oleic acid, palmitic acid, behenic acid, gadoleic acid, stearic acid, lignoceric acid, and arachidic acid. Its fatty-acid composition meets the Codex Alimentarius specifications. These data also showed no significant differences in fatty acid composition among oils produced with different raw materials and roasting temperatures. Other studies have indicated an excellent lipid profile for peanut oils, which are higher in unsaturated than in saturated fatty acids. Unsaturated fatty acids maintain the relative fluidity of cell membranes, reducing heart disease risk factors [3,7,20,27]. They may also help improve insulin sensitivity and lower blood sugar in people with diabetes [27].

Vegetable oil contains various antioxidants of great interest due to their health effects. We analyzed tocopherol in peanut oil (Table 2), which has equal quantities of α-tocopherol (38.86–55.59 μg/g) and γ-tocopherol (34.73–63.51 μg/100 g). Compared with α-tocopherol, high levels of γ-tocopherol can provide oil with better thermal oxidation stability. However, α-tocopherol has the greatest biological activity. Tocopherols are radical scavengers, delivering a hydrogen atom to quench free radicals [13,18,28]. The O–H bond in tocopherols is ~10% weaker than in most other phenols. This weak bond allows the vitamin to donate a hydrogen atom to the peroxyl radical and other free radicals, minimizing their damaging effect [28].

Phytosterols are one of the main active substances in vegetable oils that prevent cardiovascular diseases and improve the oil’s physical and chemical properties [13,15]. In this study, peanut oil contained different phytosterols. Table 2 shows that squalene (7.36–9.41 μg/g), stigmast-5-en-3-ol (7.82–8.56 μg/g), campesterol (2.71–3.28 μg/g), stigmasterol (2.41–3.11 μg/g), and stigmasta-5,24(28)-dien-3-ol (1.74–2.27 μg/g). Squalene is also considered a nutritional indicator of olive oil, a metabolic intermediate of the sterol biosynthetic pathway, and a possible target in different metabolic and oxidative stress-related disorders [15].

Phenols and flavonoids in vegetable oil have anti-inflammatory and antioxidant effects, supported by research on vegetable oils [6,11,14,29]. The potential nutritional profile of the analyzed peanut oils indicated 15.87–30.81 gallic acid equivalents (GE) μg/g total phenols and 4.17–4.91 quercetin equivalents (QE) μg/g total flavonoids (Table 2). Free radical scavenging and reducing power are important mechanisms in their antioxidant activity, indicating that the antioxidants’ electron and hydrogen atom transfers can reduce the oxidized intermediates of the lipid peroxidation process [29,30]. Antioxidant activity may vary widely depending on the hydrophobic substrate [30,31]. However, the 2,2-diphenyl-1-picrylhydrazyl (DPPH) radical scavenging activity test can effectively reflect the hydrophobic substrate performance. The results showed DPPH radical scavenging activities for P12, P14, HP12, and HP14 of 40.82, 46.21, 41.14, and 44.18%, respectively, and ferric ion reducing antioxidant power (FRAP) activities of 103.85, 220.71, 158.64, and 189.47 Trolox μg/g. This study showed that roasting degraded the tissues, destroyed bonds, released active substances, and led to the degradation of heat-sensitive components, consistent with previous studies [16,18].

### 2.2. Oxidation Stability and Volatile Generation

The Rancimat test is more suitable for edible oils than automated accelerated and regular shelf-life tests, observing volatile production during oxidation [32,33,34]. We assessed the shelf life and kinetic behavior of peanut oil with the Rancimat test. Peanut oil underwent oxidation at four different temperatures (100, 105, 110, and 115 °C) via the Rancimat test, with the induction times listed in Table 3.

The results showed that the induction time decreased with the increasing temperature. Simultaneously, a semi-logarithmic relationship for all of the oil samples by Equation I including a linear dependency with a good correlation of determination (R^2^). The Ea values for all oil samples were evaluated using Equation II. Table 3 shows that the activation energy (*E*a) values of P12, P14, HP12, and HP14 were 94.72, 90.01, 100.35, and 99.39 kJ/mol, respectively (Table 3). Oils with higher *E*a need a higher temperature to induce a specific change in the oxidation rate [34].

ln (k) = a(T) + b(1)

where a and b are the equation parameters.

ln (k) = ln (A) − (Ea/RT)(2)

where k is the reaction rate constant or reciprocal induction period (h–1), and R is the molar

The taste of peanut oil is distinct and adds a nutty flavor to any dish. However, its sensory presentation will vary based on the processing technology used [21,23]. To understand volatile formation, the Rancimat tests and HS-SPME were used to evaluate the volatile composition of peanut oil. Headspace-solid phase microextraction (HS-SPME) quantifies the composition of volatile compounds and often yields complex data [33,35]. Table 4 shows the progress of different parameters in peanut oil and the Rancimat test at 110 °C. Fifty-eight volatile compounds were detected including twenty-two *N*-heterocyclic compounds, nineteen *O*-heterocyclic compounds, five hydrocarbon compounds, five aldehyde compounds, three alcohols, and three ketones and carboxylic acids. The results show that as the degree of oxidation increases, peanut oil has a complex composition and total volatile amounts.

Characteristic volatile fingerprints are insufficient to estimate food flavors because not all volatiles are odorants, and their impacts on flavor are indirectly related to their concentration [35]. Therefore, a simple, rapid, and reliable procedure for describing important parameters such as taste and smell would be beneficial. The analysis of volatile compounds implicitly estimates the flavors of peanut oil due to the limitations of human sensory perception, which is related to volatile concentrations. The sensed compounds in peanut oil include methylpyrazine, 2,5-dimethylpyrazine, ethyl-methylpyrazine, and ethyl-dimethylpyrazine [21,22]. These compounds provide the odor of roasted peanuts. In this study, peanut oils were produced with different materials and roasting temperatures, and their flavor perception varied due to combinations of different components. The analysis of volatile compounds indicated that P12, P14, HP12, and HP14 had thirty-eight, thirty-six, thirty-two, and forty, respectively.

**Figure 1:**
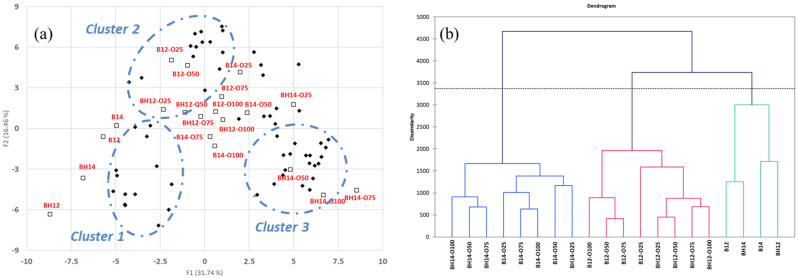
PCA (**a**) and AHC (**b**) of volatile compounds of peanut oil during the oxidation process.

During the oxidation process, various complex reactions produce different degradation products. The results show that as the degree of oxidation increases, peanut oil has a complex composition and total amount of volatiles. Since volatile generation by oxidation changes peanut oil, a principal components analysis (PCA) was used to identify clusters of aroma properties. Three main clusters were identified (Figure 1a). Cluster 1 contained those associated with the aroma from the roasting process including hexanal (green), 2-furanmethanol, 2-acetylfuran, maltol (caramellic), 1-octen-3-one, 1-octen-3-ol (earthy), benzaldehyde (almond), benzenemethanol (floral), 2-octenal, and nonanal (fatty). Cluster 2 contained those generated by the thermal oxidative degradation of volatiles and the oxidized volatiles themselves [36,37,38]. We observed the formation of phenolic derivatives (such as benzene and phenol) via the thermal degradation of lignin and benzaldehyde derivatives via phenylalanine. Cluster 3 contained oxidation volatiles participating in the Mildner reaction (such as 2-methoxy-4-vinylphenol, 2-hexylfuran, and 3-butyl-2,5-dimethylpyrazine). Previous studies have shown that advanced lipid oxidation end products are formed by non-enzymatic reactions between lipid aldehydes and amino phospholipids [39,40]. Therefore, lipid autoxidation and the Maillard reaction should not be considered as two independent reaction pathways but as a single complex interaction [37,38,40]. During agglomerative hierarchical clustering (AHC) analysis, an interesting phenomenon in peanut oil oxidation can be observed: the oxidation volatiles formed depends on the substrate and temperature differences (Figure 1b).

## 3. Materials and Methods

### 3.1. Peanut Oil Production

The peanuts (*Arachis hypogaea* L. ‘Tainan 14’) were supplied by a marketing cooperative (Chiayi, Taiwan) in February 2019. They were separated into five-kilogram batches and roasted in a roasting machine at 120 °C or 140 °C for 10 min individually. Then, the oil was extracted by pressing each batch of roasted peanuts in a pressing machine, followed by filtration and oil collection (Table 5).

### 3.2. Quality Analysis

Three oxidative tests were performed on the oil samples. The PV was determined by the official analytical methods of the European Community Regulations. The p-AV was measured using 0.25% anisidine/glacial acetic acid by UV absorbance at 350 nm. The AV was measured by titration with 0.1 N potassium hydroxide alcoholic solution.

The method followed was previously described by Ciou et al. [18].The oil sample’s color was measured using a NE-4000 colorimeter (Nippon Denshku Industries Co. Ltd.; Tokyo, Japan). First, the instrument was standardized with a white plate (L0 = 97.51, a0 = −0.16, and b0 = 1.75), and the samples were evaluated at room temperature. Next, the Hunter L, a, and b values, corresponding to lightness, greenness (^−^a) or redness (^+^a), and blueness (^−^b) or yellowness (^+^b), respectively, were inspected. Finally, the Browning index was calculated according to the following equation:

Browning index = [100 × (x − 0.312)]/0.172(3)

where x = (a + 1.75L)/(5.645L + a − 3.012b).

### 3.3. Composition Analysis

The oil sample’s fatty acid composition was evaluated via gas chromatography (GC)/flame ionization detection (FID). Triacylglycerols were converted to methyl esters using the American Oil Chemists’ Society (AOCS) Official Method Ce 2–66 [41]. Methyl esters were separated using a column coated with DB-23 (30 m × 0.25 mm × 0.25 μm) and helium as the carrier gas at a 1.0 mL/min flow rate. The oven’s temperature was initially held at 200 °C for 8 min, then increased to 220 °C at 10 °C/min before being held for 40 min. The temperatures of the FID and injector (split mode 1:40, 4 mm liner) were maintained at 270 °C and 250 °C, respectively. The fatty acid contents were determined using the normalization method. The quantitative determination of the tocopherol composition has been previously described [18]. A 20 µL aliquot of the filtrate was injected and separated using a high-performance liquid chromatography system (L-2130 pump and L-2400 UV detector; Hitachi, Japan) attached to a Mightysil RP-18GP250 column (l = 250 mm; internal diameter [i.d.] = 4.6 mm; thickness = 0.32 µm; Kanto Chemical Co. Inc., Japan). The calibration curve for each standard was established by plotting the peak area with the corresponding concentration. Phytosterol analysis was performed by GC/mass spectrometry (MS) as described previously using Agilent instruments equipped with a DB-1 column (60 m × 0.25 mm i.d.; Agilent, CA, USA) [42]. The 5α-cholesterol was used as an internal standard, and the ratio of the peak areas of the analyte and the internal standard was used as the analytical signal. Total phenolics and total flavonoids were analyzed by UV spectrophotometry. Each oil sample (0.5 g) was diluted with acetone to a volume of 20 mL. The total phenolic compound contents were expressed as GE using the Folin–Ciocalteu reagent [18]. The total flavonoid content was expressed as QE/g using the aluminum chloride colorimetric method [18]. The calibration curve for each standard was calculated by plotting its peak area with its corresponding concentration.

### 3.4. Antioxidant Activity

The antioxidant activity was measured using the DPPH and FRAP assays. The DPPH radical-clearing capacity was measured using a previously published reference [18]. First, each oil sample was diluted to 1 mg/mL with a solution of acetone/methanol (2:8) and then mixed with DPPH radicals (0.2 mM) in a methanol solution. After vigorous shaking, the mixtures were incubated at room temperature for 30 min, and their UV absorbance at 517 nm was measured. FRAP was measured as previously described [18]. Each oil sample was diluted with acetone/methanol (2:8) solution to 2 mg/mL and then mixed with FRAP reagent (acetate buffer, iron chloride solution, and 2,4,6-tris [2-pyridyl]-s-triazine; 10:1:1). After vigorous vortexing, the mixture was incubated at room temperature for 10 min, and its UV absorbance at 595 nm was measured. Trolox was used as the positive control.

### 3.5. Rancimat Test Kinetic Parameter

The oxidative stability of the tested oil samples (5 g) was assessed at different temperatures (100, 105, 110, and 115 °C) using the Rancimat 743 apparatus. The induction period (h) and the critical test points were automatically recorded at an airflow rate of 10 L/h. The intersect point of the two extrapolated curves was taken as the induction period for each sample. The kinetic parameters were based on a previously described method [33] with slight modification. The kinetic rate constant, temperature coefficients (T Coeff; K^−1^), *E*a (kJ/mol), and pre-exponential or frequency factors (A; h^−1^) were defined as previously described [16].

### 3.6. Volatile Compound Analysis

HS-SPME analysis was used to understand the volatile compound characteristics. The method followed was previously described by Yang et al. [21]. During the sampling process, a 50/30 μm divinylbenzene/carboxen/polydimethylsiloxane (DVB/CAR/PDMS) fiber was inserted into the reaction vessel, and the sample was kept under Rancimat testing conditions for 20 min. Then, the fiber was removed from the reaction vessel and inserted into the GC/MS injector to desorb the compounds. The contents were kept in the GC/MS injector for 15 min at 250 °C, and Agilent (Santa Clara, CA, USA) Model 7890 GC was attached to an Agilent Model 5977A detector using an Agilent DB-1 (60 m × 0.25 mm i.d.). Helium acted as the carrier gas at a 1 mL/min flow rate. The injection port temperature was set at 250 °C, the ion source temperature at 230 °C, and the ionization potential was 70 eV. The GC oven temperature program was set at 40 °C for 1 min, followed by 5 °C/min increments up to 150 °C, where it was held for 1 min before 10 °C/min increments up to 200 °C, where it was held for 11 min. The linear refractive indices were calculated from the retention times of n-alkanes (C_5_–C_25_) performed under the same chromatographic conditions.

### 3.7. Statistical Analysis

All experiments were conducted in triplicate and expressed as the mean ± standard deviation. The data were subjected to an AHC with squared Euclidean distances. Then, the results were analyzed using PCA and Varimax rotation. The AHC and PCA analyses were performed with the XLSTAT software (version 2010.2.01, Addinsoft Deutschland, Andernach, Germany).

## 4. Conclusions

These results showed that the edible oil produced by *Arachis hypogaea* L. (Tainan No. 14) was in accordance with the standard of the edible oil quality. PCA and AHC analysis indicated that processing parameters such as shelling treatment and roasting temperatures mainly affected the chemical composition and volatile formation. Tainan No. 14 has the potential for functionality and deserves further investigation in the future.

## Figures and Tables

**Table 1:** Quality index and fatty acid composition (%) for P12, P14, HP12, and HP14.

	P12	P14	HP12	HP14
	Quality indices			
Yield	32.55	33.61	29.78	30.13
BI	26.54	25.67	26.63	26.26
PV	5.61	6.13	6.32	6.88
AV	1.25	1.11	1.65	0.98
p-AV	6.15	12.63	6.23	10.66
TOTOX	17.35	24.86	20.63	24.42
	Fatty acid compositions (%)			
C16:0	14.03	13.44	13.88	13.04
C18:0	1.78	1.62	1.63	1.64
C18:1	37.70	41.41	37.84	41.68
C18:2	40.69	38.86	41.29	38.40
C20:0	0.94	0.77	0.91	0.82
C20:1	1.31	1.19	1.36	1.33
C22:0	2.43	1.96	2.18	2.16
C24:0	1.12	0.77	0.91	0.92

Data presented are in mean ± SD form (n = 3). BI, browning index; PV, peroxide value (meq/kg); AV, acid value (mg KOH/g); p-AV, anisidine value (meq/kg); TOTOX, total oxidation value (meq/kg).

**Table 2:** The antioxidant composition and antioxidant activity for P12, P14, HP12, and HP14.

	P12	P14	HP12	HP14
	Antioxidant Components			
γ-tocopherol ^1^	56.15 ± 2.36 ^c^	43.67 ± 2.28 ^b^	63.51 ± 3.80 ^d^	34.73 ± 1.07 ^a^
α-tocopherol ^1^	55.59 ± 1.95 ^c^	48.65 ± 2.21 ^b^	52.41 ± 1.64 ^b^	38.86 ± 2.29 ^a^
squalene ^1^	7.36 ± 0.20 ^a^	8.25 ± 0.67 ^a^	9.41 ± 0.33 ^a^	8.11 ± 0.55 ^a^
campesterol ^1^	2.89 ± 0.07 ^a^	2.91 ± 0.15 ^a^	3.28 ± 0.21 ^a^	2.71 ± 0.28 ^a^
stigmasterol ^1^	2.58 ± 0.21 ^a^	2.69 ± 0.24 ^a^	3.11 ± 0.18 ^a^	2.41 ± 0.12 ^a^
stigmast-5-en-3-ol ^1^	8.56 ± 0.71 ^a^	8.23 ± 0.60 ^a^	7.82 ± 0.64 ^a^	7.91 ± 0.17 ^a^
stigmasta-5,24(28)-dien-3-ol ^1^	1.74 ± 0.30 ^a^	1.80 ± 0.32 ^a^	2.27 ± 0.33 ^a^	2.15 ± 0.47 ^a^
Total phenol ^2^	19.30 ± 1.69 ^b^	30.81 ± 2.61 ^c^	15.87 ± 1.75 ^a^	20.36 ± 1.42 ^b^
Total flavonoid ^3^	4.17 ± 0.33 ^a^	4.38 ± 0.32 ^ab^	4.59 ± 0.41 ^b^	4.91 ± 0.21 ^b^
	Antioxidant activity			
DPPH ^4^	40.82 ± 3.15 ^a^	46.21 ± 2.91 ^a^	41.14 ± 2.15 ^a^	44.18 ± 2.48 ^a^
FRAP ^5^	103.85 ± 6.61 ^a^	220.71 ± 10.92 ^d^	158.64 ± 9.62 ^b^	189.47 ± 11.74 ^c^

Data presented are in mean ± SD form (n = 3), ^a–d^ with different letters indicating values that are significantly different at *p* < 0.05. ^1^ μg/g; ^2^ GE μg/g; ^3^ QE μg/g; ^4^ %; ^5^ TrE μg/g.

**Table 3:** Regression parameters for the Arrhenius relationships between the reaction rate constant and the temperature for P12, P14, HP12, and HP14.

	P12	P14	HP12	HP14
	Induction time (hours)			
100 °C	16.18	17.12	15.78	19.48
105 °C	10.8	12.31	10.77	14.77
110 °C	8.77	9.1	7.02	10.67
115 °C	4.66	5.36	4.53	5.46
	ln(K) = a(1/T) + b			
a	−11.39	−10.93	−12.07	−11.95
b	27.73	26.45	29.58	28.99
*E*a	94.72	90.01	100.35	99.39

**Table 4:** The volatile compounds identified in peanut oil during the oxidation process via HS-SPME and GC/MS.

Peak	RI	Compounds
1	800	hexanal
2	812	methylpyrazine
3	829	2-furanmethanol
4	881	2-acetylfuran
5	886	2,5-dimethylpyrazine
6	893	ethenylpyrazine
7	931	benzaldehyde
8	954	2,2-dimethoxyethanol
9	957	1-octen-3-one
10	964	1-octen-3-ol
11	980	ethyl-methylpyrazine
12	992	ethenyl-methylpyrazine
13	1002	benzenemethanol
14	1008	benzeneacetaldehyde
15	1022	2-acetylpyrrole
16	1032	2-octenal
17	1036	acetophenone
18	1052	1-octanol
19	1061	ethyl-dimethylpyrazine
20	1066	2,5-diethylpyrazine
21	1074	2-methyl-5-propenylpyrazine
22	1079	maltol
23	1082	nonanal
24	1087	2-acetyl-3-methylpyrazine
25	1091	benzyl nitrile
26	1100	undecane
27	1103	methyl nicotinate
28	1110	5-methyl-6,7-dihydro-5H-cyclopenta[b]pyrazine
29	1122	2-butyl-3-methylpyrazine
30	1129	1-ethenyl-4-methoxybenzene
31	1137	diethyl-methylpyrazine
32	1139	4-ethylphenol
33	1155	2,4,6-trimethylbenzaldehyde
34	1164	2-methyl-5H,6,7-dihydrocyclopentapyrazine
35	1172	2-decanone
36	1176	2-pentylpyridine
37	1184	2,5-dimethyl-3-(2-methylpropyl)pyrazine
38	1187	2,3-dihydrobenzofuran
39	1193	2-acethyl-3,5-dimethylpyrazine
40	1195	3,5-dimethyl-2-(Z-1-propenyl)-pyrazine
41	1200	dodecane
42	1201	2-methyl-1,3-cyclohexanedione
43	1204	4-Oxononanal
44	1228	2-methyl-6-(3-methyl-butyl)-pyrazine
45	1234	5-pentyl-3H-furan-2-one
46	1238	2-phenyl-2-butenal
47	1257	4-ethyl-2-methoxyphenol
48	1260	formamidobenzene
49	1271	2,4-decadienal
50	1289	2-methoxy-4-vinylphenol
51	1290	2-hexylfuran
52	1295	3-butyl-2,5-dimethylpyrazine
53	1300	tridecane
54	1312	methyl-2-(1-methyl-2-pyrrolidinylidene)acetate
55	1322	dihydro-5-pentyl-2(3H)-furanone
56	1400	tetradecane
57	1416	tridecanoic acid
58	1458	3-eicosene

RI: Retention index, identified via comparison of the mass spectra with the RI; RI obtained from the literature.

**Table 5:** The peanut oil sample preparation and its abbreviation.

Sample Name	Description
P12	Unshelled peanuts roasted at 120 °C
P14	Unshelled peanuts roasted at 140 °C
PH12	Shelled peanuts roasted at 120 °C
PH14	Shelled peanuts roasted at 140 °C

## Data Availability

Not applicable.

## Sample Availability

Samples of the compounds are not available from the authors.
